# Neonatal pain assessment and non-pharmacological analgesia: an integrative literature review

**DOI:** 10.3389/fpain.2026.1820044

**Published:** 2026-07-16

**Authors:** Ronghua Yang, Xia Zhang

**Affiliations:** Department of Neonatology, The First Affiliated Hospital of Hunan Traditional Chinese Medical College (Hunan Provincial Directly Affiliated Hospital of Traditional Chinese Medicine), Zhuzhou, China

**Keywords:** artificial intelligence, clinical practice, kangaroo care, neonatal pain, non-pharmacological analgesia, pain assessment tools

## Abstract

**Background:**

Neonatal procedural and prolonged pain remains a frequent and clinically important problem in neonatal care, particularly among preterm and critically ill infants who undergo repeated invasive procedures.

**Objective:**

To synthesize evidence on neonatal pain assessment tools and non-pharmacological analgesic strategies, with attention to clinical implementation and emerging objective or digital assessment approaches.

**Methods:**

PubMed, CINAHL, MEDLINE, the Cochrane Library, Web of Science, Scopus, and Embase were searched for peer-reviewed English-language articles published from January 2000 to May 2025. Eligible studies included primary research, high-quality secondary evidence, and clinically relevant methodological or technological studies involving preterm or term neonates, defined as infants with a birth age of ≤28 days, or infants cared for in neonatal intensive care units. Studies were included if they addressed the development, validation, or clinical application of neonatal pain assessment tools, or the efficacy, safety, or implementation of non-pharmacological analgesic interventions. Studies limited to pharmacological analgesia, older children, or adults were excluded.

**Results:**

Thirty-five studies were included. The evidence supports the use of validated multidimensional assessment tools for structured evaluation of neonatal pain. It indicates that family-integrated, sensory, and behavioral non-pharmacological approaches can reduce procedural pain responses. However, the strength and consistency of evidence are greater for single procedures than for repeated or prolonged pain. Artificial intelligence-based facial expression analysis, physiological monitoring, and biomarker-based approaches show potential for more objective pain detection but remain insufficiently validated for routine bedside decision-making.

**Conclusion:**

Standardized neonatal pain management should combine validated assessment scales, protocolized first-line non-pharmacological care, staff training, and parent involvement. Future research should prioritize repeated and prolonged pain, high-risk neonatal populations, implementation in routine neonatal intensive care practice, and the prospective validation of digital assessment systems in real-world clinical settings.

## 1 Introduction

Accurate pain assessment and effective management of procedural and persistent pain remain major unresolved challenges in neonatal intensive care. In the past, the inability of newborns to express pain verbally, together with the outdated belief that their nervous system is too immature to process pain, led to insufficient attention being paid to neonatal pain (1). As a result, developing a systematic, evidence-based framework for pain assessment and relief has become an urgent clinical priority. In recent years, non-pharmacological analgesic approaches have drawn growing clinical interest because of their excellent safety profiles and ready availability (2, 3). At the same time, technological advances—particularly artificial intelligence (AI)—have offered entirely new ways to achieve more objective and continuous pain assessment (4, 5). The present integrative review systematically synthesizes recent research findings, with the goal of offering a practical yet scientifically rigorous guidance framework for the assessment and management of neonatal pain.

Preterm neonates are especially susceptible to the harmful effects of repeated procedural pain. Due to the immaturity of descending inhibitory pathways and the inability to mount effective endogenous analgesic responses, preterm infants experience pain at lower thresholds and with greater intensity than term infants (6, 7). A growing body of work suggests unaddressed or not well controlled pain during NICU stay is associated with disrupted brain development, including reduced white matter integrity, decreased subcortical gray matter volumes, and abnormal maturation of the somatosensory cortex (8, 9). Beyond structural changes, repeated pain exposure has been linked to HPA axis imbalance, leading to altered cortisol reactivity patterns that may persist beyond the neonatal period (7, 10). Clinically, these neurobiological alterations translate into long-term behavioral abnormalities, such as hyperalgesia, heightened stress responses, attention deficits, and internalizing disorders in childhood and adolescence (6, 9). Also, early pain exposure may interfere with mother-infant bonding and increase parental distress, creating a cycle gthat further compromises developmental outcomes (9, 11). Given these risks, there's a clear need for effective, standardizable, and low in risk pain management strategies made specifically for preterm infants. To address this demand, researchers have come up with various validated pain assessment tools and put them into practice in clinical practice.

Currently, a variety of validated pain assessment tools are available in clinical practice, which are primarily categorized into two types—multidimensional and unidimensional—based on the number of assessment dimensions. Among these, multidimensional scales integrate multiple indicators, including behavioral, physiological, and contextual aspects, enabling comprehensive and systematic assessment. Therefore, they are often recommended for various clinical scenarios and can fully exert their advantages in complex disease conditions or intensive care settings. Commonly used and well-investigated multidimensional tools include the Neonatal Infant Pain Scale (NIPS) (12), the Premature Infant Pain Profile (PIPP) (12, 13), and the Neonatal Facial Coding System (NFCS), as well as its revised version (NFCS-R) (12, 14). Research has confirmed that these tools possess good reliability and validity in distinguishing painful from non-painful stimuli (14). Unidimensional tools focus on a single assessment domain, such as the Children and Infants’ Postoperative Pain Scale (CHIPPS). Despite their single-dimensional assessment scope, they still have considerable application value in specific scenarios, such as postoperative pain (14). However, regardless of the tool selected, a comprehensive judgment must be made based on the neonate's gestational age, clinical condition, and type of pain (acute procedural, prolonged, or postoperative) (15).

A substantial body of research evidence confirms that a variety of non-pharmacological interventions yield significant effects in alleviating procedural pain in neonates. Kangaroo Care (Skin-to-Skin Contact) can consistently reduce neonatal pain scores (e.g., Premature Infant Pain Profile [PIPP], Neonatal Infant Pain Scale [NIPS]), heart rate, crying duration, and levels of stress hormones such as cortisol during clinical procedures, including heel lance and venipuncture (9, 16–18). In addition, this nursing intervention can alleviate NICU-related stress responses in neonates and help them maintain physiological stability (17, 19). Initiating breastfeeding during clinical procedures exerts an analgesic effect through a multisensory synergistic mechanism involving sucking, taste, olfaction, and physical holding, with definite and applicable efficacy for both term and late-preterm infants (20–22). Oral administration of expressed human milk also has an analgesic effect; relevant studies have shown that its analgesic efficacy is comparable to that of 24% sucrose solution in some neonatal populations (23, 24). Oral sucrose or glucose remains a non-pharmacological analgesic intervention with the most thorough research and a definite effect in single painful procedures (25, 26). However, there is insufficient evidence to support the analgesic efficacy and safety of such solutions for repeated procedural pain. Caution should be exercised in clinical application, and further in-depth research is warranted (27, 28). Providing a pacifier for neonates to suck on during clinical procedures (Non-Nutritive Sucking, NNS) can distract their attention and provide physical comfort, thereby effectively reducing pain-induced behavioral responses (2, 29). Physical containment techniques such as Facilitated Tucking and Swaddling can provide postural support and a comforting swaddling sensation for neonates, significantly lowering their pain scores and reducing pain-related motor agitation (11, 30). Multisensory combined strategies integrating two or more non-pharmacological analgesic interventions (e.g., swaddling combined with oral sucrose, breastfeeding combined with skin-to-skin contact, non-nutritive sucking combined with gentle rocking) generally yield superior analgesic effects compared with single interventions (30–34). In addition, sensory saturation interventions and clinical environmental modification measures (e.g., fitting neonates with eye shields and ear muffs to reduce light and sound stimulation) have demonstrated promising potential for application in neonatal pain management (35).

Emerging technologies have also advanced neonatal pain assessment: Studies have shown that healthcare providers’ visual attention areas exhibit dynamic characteristics during neonatal pain assessments. After a clinical procedure, their attention to the neonates’ lower facial region and upper limbs increases significantly, and this pattern of visual attention is significantly correlated with pain perception (36, 37). Machine learning models, especially those that analyze facial expressions using convolutional neural networks (CNNs), can achieve highly accurate classification and recognition of neonatal pain states from video data (5, 38, 39). However, the clinical application scope of this technology remains limited at this stage due to constraints such as facial occlusion, lighting variability, and the need for large, diverse datasets (4). Currently, AI-driven tools capable of real-time output of continuous “pain signature signals” are in the research and development stage (5). The academic community continues to explore objective correlative indicators that can reflect pain states, including fluctuations in cerebral regional oxygen saturation (CrSO₂) detected via near-infrared spectroscopy (NIRS) (18, 40), changes in skin blood flow (25), and variations in the expression of hormonal biomarkers such as salivary cortisol (10, 17).

Nevertheless, the available literature remains fragmented across several domains, including assessment scales, bedside non-pharmacological interventions, biomarkers, and AI-assisted technologies. Many reviews summarize efficacy findings, but fewer integrate the methodological strengths and limitations of these bodies of evidence or consider how feasibility, staff training, parental participation, and NICU workflow influence implementation. Therefore, this integrative review was designed to answer a focused clinical question: how can validated pain assessment approaches and feasible non-pharmacological analgesic strategies be integrated into standardized neonatal pain management, and what evidence gaps limit their translation into routine practice?

## 2 Materials and methods

### 2.1 Review questions and PICOS framework

The review question was structured using a PICOS framework: (1) P (Population): Preterm and term neonates, defined as infants with a birth age of ≤28 days, and infants cared for in neonatal intensive care units; (2) I (Intervention): Neonatal pain assessment tools, non-pharmacological analgesic interventions, and emerging objective or digital approaches for pain assessment; (3) C (Comparator): Usual care, placebo, no intervention, alternative non-pharmacological interventions, or clinician-based assessment, depending on the design of the original study; (4) O (Outcomes): Pain scores, behavioral responses, physiological responses, crying duration, stress-related biomarkers, feasibility, safety, and implementation considerations; (5) S (Study design): Randomized or quasi-experimental trials, observational studies, qualitative studies, systematic reviews, meta-analyses, and clinically relevant methodological studies.

### 2.2 Inclusion and exclusion criteria

Articles were included if they met the following criteria: (1) published in English in a peer-reviewed journal; (2) published between January 2000 and May 2025; (3) available as full-text primary research studies, including randomized controlled trials, quasi-experimental studies, cohort studies, case-control studies, observational studies, or qualitative studies, or as high-quality secondary research studies, including systematic reviews and meta-analyses; (4) focused on preterm or term neonates, defined as infants with a birth age of ≤28 days, or infants cared for in neonatal intensive care units; and (5) addressed the development, validation, or application of neonatal pain assessment tools, the efficacy or safety of non-pharmacological analgesic interventions, or emerging objective or digital approaches for neonatal pain assessment.

Articles were excluded if they met any of the following criteria: (1) publications not written in English; (2) non-research publications, including editorials, letters to the editor, case reports, dissertations, conference abstracts, conference summaries, and expert opinions; (3) studies focusing on participants older than 28 days, children beyond the neonatal period, or adults; (4) studies concentrating exclusively on pharmacological analgesic interventions without any assessment of non-pharmacological approaches; and (5) studies with insufficient methodological quality, such as systematic reviews that did not report PRISMA-based methods or randomized trials without clear descriptions of randomization procedures or allocation methods.

### 2.3 Literature search

A structured search was conducted in PubMed, MEDLINE (Ovid), CINAHL (EBSCO), the Cochrane Library, Web of Science, Scopus, and Embase for articles published from January 2000 to May 2025. The search combined controlled vocabulary and free-text terms related to neonates and pain, including “neonate”, “newborn”, “preterm infant”, “NICU”, “neonatal pain”, “pain assessment”, “pain scale”, “procedural pain”, “non-pharmacological analgesia”, “kangaroo care”, “skin-to-skin”, “breastfeeding”, “sucrose”, “non-nutritive sucking”, “swaddling”, “facilitated tucking”, “artificial intelligence”, and “machine learning”. Boolean operators (AND/OR) were used, and searches were adapted to each database's syntax. Titles, abstracts, and keywords were screened, and the reference lists of relevant reviews and clinical guidelines were manually checked to identify additional studies.

### 2.4 Study selection process

**Figure 1:**
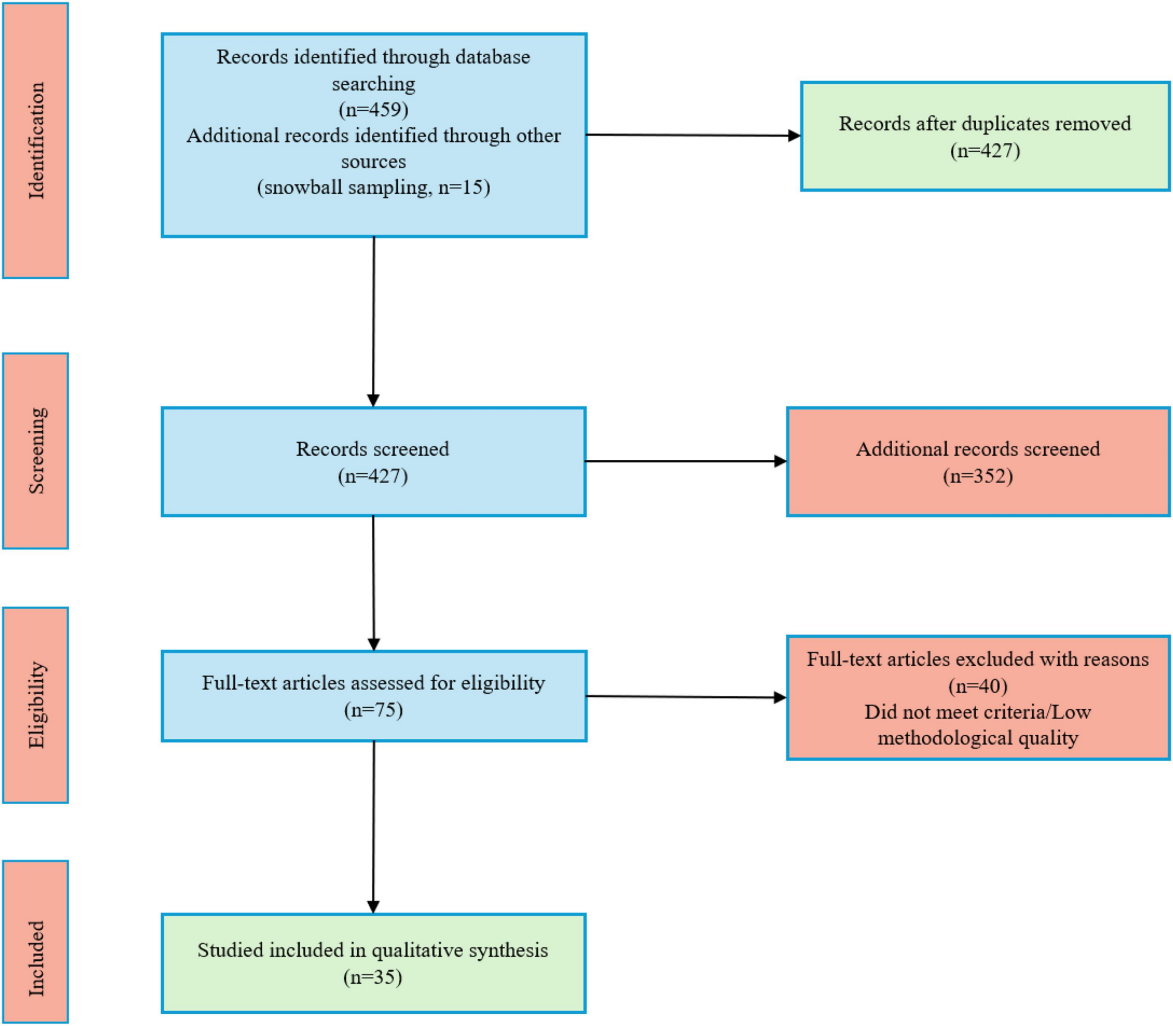
The PRISMA flow chart.

The study selection process followed the PRISMA reporting framework (41). All records were imported into a reference-management file, and duplicates were removed before screening. Two reviewers (R.Y and X.Z) independently screened titles and abstracts against the eligibility criteria, followed by full-text assessment of potentially relevant articles; disagreements were resolved through discussion. The initial database search identified 459 articles, and 15 additional articles were retrieved through reference-list screening. After duplicate removal (*n* = 47), 427 records remained for title and abstract screening. Seventy-five articles were assessed in full text, and 35 articles were included in the qualitative synthesis. The main reasons for exclusion at full-text review were non-neonatal populations, a pharmacological-only focus, insufficient information on assessment or non-pharmacological outcomes, duplicate or overlapping evidence, and inadequate methodological quality (Figure 1).

### 2.5 Data extraction, methodological appraisal, and synthesis

For each included study, information was extracted on study design, population characteristics, gestational age or clinical setting, type of pain or procedure, assessment tool or intervention, comparator, outcome measures, main findings, and implementation considerations. Because the included literature comprised heterogeneous primary studies, systematic reviews, and technological-methodological studies, a narrative integrative synthesis was performed following the methodological principles of Whittemore and Knafl (42). The strength of evidence was interpreted according to study design, sample size, consistency of results, clarity of outcome measurement, and risk of bias or methodological limitations. Findings were grouped into three domains: pain assessment tools, non-pharmacological analgesic interventions, and emerging digital or biomarker-based approaches.

## 3 Results

### 3.1 Landscape of pain assessment practices

Based on the included studies, 7 commonly used neonatal pain assessment tools were sorted out, including 3 behavioral scales, 3 composite (multidimensional) scales, and 1 global clinical rating scale. These tools apply to preterm and full-term neonates, and cover the assessment of acute procedural pain, prolonged pain, and postoperative pain. The behavioral scales focus on observable behavioral indicators (e.g., facial expressions, crying, body movements, and postures), such as the Neonatal Infant Pain Scale (NIPS), Neonatal Facial Coding System (NFCS & NFCS-R), and Children and Infants’ Postoperative Pain Scale (CHIPPS). The composite scales integrate multiple assessment dimensions, including behavioral, physiological, and contextual factors, such as the Premature Infant Pain Profile (PIPP), Scale for Use in Newborns (SUN), and Neonatal Pain, Agitation and Sedation Scale (N-PASS). Additionally, the Visual Analog Scale/Numerical Rating Scale (VAS/NRS) serves as a global clinical rating tool for evaluating subjective pain intensity (Table 1).

**Table 1:** Summary of neonatal pain assessment tools.

Tool name (abbreviation)	Key features/indicators	Intended population & context	Psychometric properties/key findings (from provided references)	Clinical applicability/feasibility	Key limitations
Neonatal infant pain scale (NIPS) (12, 37)	Assesses five behavioral factors (facial expression, cry, arms, legs, state of arousal) and one physiological factor (breathing pattern).	Preterm and full-term neonates; acute procedural and postoperative pain.	Used in studies to compare pain response to devices (43) and interventions (44). High inter-rater reliability reported (14).	Very high clinical feasibility (quick scoring, no equipment); among the most widely used tools in routine NICU practice (5).	Does not directly measure pain intensity; limited utility for prolonged pain or ventilated infants (5).
Premature infant pain profile (PIPP) (12, 13)	Multidimensional scale incorporates three behavioral dimensions (facial actions) as well as two physiological indices (heart rate, oxygen saturation) and two contextual indicators (gestational age, behavioral state)	Preterm and term newborn infants; procedural acute pain.	Common outcome measure in meta-analyses on non-pharmacological interventions (13, 26). Revised version (PIPP-R) used in device comparison trials (40).	Well validated for preterm and term infants; moderate feasibility (requires GA calculation and baseline state assessment) (14).	Physiological parameters may reduce internal consistency; less suitable for prolonged/chronic pain (8).
Neonatal facial coding system (NFCS) & revised (NFCS-R) (12, 14)	Detailed, objective coding system for specific facial actions (e.g., brow bulge, eye squeeze, nasolabial furrow).	Preterm and full-term neonates; acute pain stimuli.	Considered to have pain evidence based on brain activity, used as a gold standard for training machine learning models (38). Compared favorably with CHIPPS in psychometric properties (14).	High content validity for facial pain expression; well suited for research and validation studies (5).	Requires extensive coder training (up to 8 h); not a standalone clinical pain assessment tool; does not incorporate physiological or contextual indicators (5).
Children and infants’ postoperative pain scale (CHIPPS) (14)	Five behavioral indicators: crying, facial expressions, trunk posture, leg posture, and motor restlessness.	Infants and toddlers; postoperative pain.	Demonstrated high inter-rater reliability and internal consistency. Psychometric performance was comparable to that of the NFCS-R (14).	Moderate feasibility; specifically designed for postoperative pain (14).	Not validated for acute procedural pain in preterm infants; limited to postoperative setting (14).
Scale for use in newborns (SUN) (7)	Assesses seven dimensions: central nervous system state, breathing, movement, tone, face, heart rate, and mean blood pressure.	Preterm and term newborn infants; procedural acute pain.	Listed among identified multidimensional scales suitable for preterm and full-term infants (7).	Limited feasibility data; requires physiological monitoring (7).	Complexity may limit routine bedside use; fewer validation studies compared to PIPP/NIPS (7).
Neonatal pain, agitation and sedation scale (N-PASS) (7)	Assesses crying/irritability, behavior/state, facial expression, extremities/tone, and vital signs (HR, RR, BP, SpO2).	Preterm and full-term neonates; assesses both pain and sedation, suitable for prolonged pain.	Listed as a multidimensional tool for assessing acute procedural pain (7).	Excellent for ventilated infants; applicable across multiple pain types; highest nurse preference in complex NICU settings (48).	Complexity may limit use in resource-limited settings; requires some training (48).
Visual analog scale/numerical rating scale (VAS/NRS) (36)	Clinician's overall rating of pain intensity on a scale (e.g., 0–10 or 0–100 mm).	Used by clinicians as an intuitive global assessment.	Used to study the correlation between clinicians’ gaze patterns and pain intuition (36, 37). Often serves as a comparison for validated tools.	Very high feasibility (no training required) (36).	Highly subjective; not validated as a standalone neonatal pain assessment tool; should not replace structured scales (36).

No single pain assessment scale for newborns has been universally recognized as the “gold standard” (3, 12). When selecting a pain assessment scale, factors such as the clinical context (acute procedural pain versus long - term/post - operative pain), the gestational age and condition of the infants, the setting (research environment vs. bedside care), and the resources available for rater training should be considered. For infants requiring respiratory support, the N - PASS may offer superior feasibility and clinical utility (48). For research - oriented coding of facial pain expressions, the NFCS remains a well - validated and reliable reference method, but it should not be described as the clinical gold standard for overall pain assessment.

While validated tools exist, their application can be inconsistent. Clinical intuition still plays a role, though studies show it aligns moderately with structured tool scores. The psychometric properties of tools like NFCS-R and CHIPPS are comparable, making practical considerations like ease of use and training requirements important factors in selection (14).

### 3.2 Efficacy of non-pharmacological interventions

Based on the included studies, five categories of non-pharmacological analgesic interventions were sorted out. These interventions can effectively alleviate neonatal pain, and combining multiple interventions often yields a stronger analgesic effect (Table 2).

**Table 2:** Summary of neonatal Non-pharmacological analgesic interventions.

Intervention category	Specific intervention	Proposed mechanism/key features	Key evidence from references (efficacy & considerations)
Skin-to-skin contact	Kangaroo Care (KC)/Skin-to-Skin Care (SSC)	Thermal, tactile, and olfactory stimulation promote physiological regulation and reduce stress hormone levels.	Strong evidence. Consistently reduces pain scores (PIPP, NIPS), crying time, and physiological stress (9, 16, 17). Reduces cortical pain responses on NIRS (18). Effective for both single procedures and reducing NICU stress (17, 19, 45).
Oral nutritive & sweet solutions	Breastfeeding (Direct)	Multisensory: suckling, taste (lactose/fat), smell, skin contact, holding.	Highly effective for term/late-preterm infants. Superior or non-inferior to sucrose in reducing pain scores (20, 21, 23, 29). Associated with specific cortical activation patterns (46)
	Expressed Breast Milk (EBM)	Taste (sweet), smell, and gut-mediated effects.	Effective alternative. Shown to reduce pain compared to control; considered a safe and natural option (13, 24).
	Sucrose/Glucose Solutions	Sweet taste-induced endogenous opioid release.	Well-established for single procedures. Effectively reduces behavioral pain scores and crying (25, 26). Efficacy for repeated procedures is less clear, requiring cautious use (27, 28).
Sensory & behavioral strategies	Non-Nutritive Sucking (NNS)	Sucking provides distraction, organizes behavior, and may stimulate orogastric vagal activity.	Effective adjuvant. Reduces crying and behavioral distress during procedures (2, 29). Often used in combination.
	Facilitated Tucking/Swaddling	Containment provides postural support, tactile comfort, and reduces motor agitation.	Effective for procedural pain. Reduces pain scores and promotes self-regulation (11, 30). Foundation for combined interventions.
	Olfactory Stimulation (e.g., Breast Milk Odor)	Familiar, pleasant odor provides comfort and counteracts noxious stimuli.	Promising evidence. Maternal breast milk odor has been shown to have a calming effect and reduce pain scores (20, 47).
	Sensory Reduction (Eye Shields/Ear Muffs)	Reduces environmental light and sound, lowering sensory overload and stress.	Emerging evidence. Combined use has been shown to lower pain intensity during venipuncture (35).
Combined/multisensory Interventions	Bundles of Care (e.g., SSC + NNS, Swaddling + Sucrose + Music)	Synergistic effect by addressing multiple sensory pathways simultaneously.	Superior efficacy. Multiple studies show combinations (2 + interventions) are more effective than single modalities (30–34). Recommended for optimal analgesia.
Parent-integrated care	Parental Holding, Rocking, Soothing Voice	Familiarity, security, and distraction are provided by the primary caregiver.	Core component of family-centered care. Integral to interventions like KC and breastfeeding. Direct holding/containment by parents is analgesic (46).

In terms of the evidence hierarchy, kangaroo care and breastfeeding are clearly proven to be highly effective, non-invasive first-line interventions. Sweet solutions have a definite analgesic effect on single procedural pain, yet their use for repeated procedural pain requires strict adherence to standardized protocols. Multimodal combined interventions consistently yield the optimal outcomes and are the preferred clinical approach. Meta-analyses have also verified the effectiveness of these non-pharmacological interventions, which can significantly reduce neonatal pain scores and crying duration. However, their effects on physiological indicators such as heart rate and oxygen saturation exhibit certain variability (13, 48). Overall, the evidence most consistently supports family-integrated and sensory-behavioral strategies for acute procedural pain, particularly when interventions are standardized and delivered before or during painful procedures. Sweet-tasting solutions have a well-established analgesic effect for single procedures, but evidence regarding frequent repeated exposure and long-term safety remains less certain. Combined or multimodal interventions may offer additive benefits, although comparisons across studies are complicated by variation in intervention components, timing, dose, and pain scales. Thus, these interventions should be interpreted as core components of a broader pain-prevention protocol rather than interchangeable stand-alone techniques (13, 48, 49).

### 3.3 Limitations of AI and physiological monitoring

In the controlled experimental setting of single-frame analysis, the performance of artificial intelligence-based pain classification models in assessments outperforms that of healthcare providers (38). However, a notable gap still exists between the actual performance of current algorithms and the clinical bedside requirements for real-time assessment tools with high stability (4). Physiological indicator monitoring can provide objective data with complementary value, yet the specificity of this approach remains inadequate, making it currently infeasible for use as an independent method in pain diagnosis.

AI-based facial expression analysis and machine-learning models show promise under controlled experimental conditions, and some studies report classification accuracy similar to, and in some cases better than clinician judgment. However, most models remain in the development or early-validation stage. Their bedside utility is held back by facial occlusion, variable lighting, positioning, small or non-diverse datasets, and the need for explainable outputs that clinicians can interpret. Physiological and biomarker-based monitoring may complement behavioral scales, but current indicators aren't specific enough to stand alone as diagnostic tools for neonatal pain.

## 4 Discussion

We put together what's known about pain assessment tools in newborns, non-drug pain relief methods, and newer digital or objective approaches What we found is that validated multidimensional pain assessment tools, PIPP and NIPS, remain clinically useful because they incorporate behavioral, physiological, and contextual indicators and are applicable across different neonatal care scenarios. However, this paper focuses on the Premature Infant Pain Profile (PIPP) and the Neonatal Infant Pain Scale (NIPS) for two main and interrelated reasons: (1) These two scales are the most widely used and highly recognized assessment tools in routine clinical work for evaluating acute procedural pain in the neonatal intensive care unit; (2) Studies so far suggest that both scales possess good psychometric properties. Specifically, the validity of the Premature Infant Pain Profile and its revised version (PIPP/PIPP - R) is rated as “excellent”, with an inter - rater reliability coefficient ranging from 0.93 to 0.96 (13); the Neonatal Infant Pain Scale (NIPS) has high internal consistency (Cronbach's alpha coefficient reaching a maximum of 0.95) and excellent inter - rater reliability (Pearson's correlation coefficient ranging from 0.92 to 0.97) (2, 14). That's not to say that PIPP and NIPS are absolutely superior to other scales in all evaluation dimensions. Other scales, such as the Neonatal Pain, Agitation, and Sedation Scale (N - PASS) and the Neonatal Facial Coding System (NFCS), do better in certain situations (e.g., assessment of persistent pain, scoring based solely on facial expressions) (7, 48). This paper emphasizes these two scales because they combine excellent psychometric performance with clinical practicality, a characteristic that is particularly useful for routine bedside assessments. The review also shows that several non-pharmacological interventions, including kangaroo care, breastfeeding or breast milk, oral sucrose or glucose for single procedures, non-nutritive sucking, facilitated tucking, swaddling, and combined multisensory strategies, can reduce neonatal procedural pain responses. In addition, emerging technologies such as facial-expression-based artificial intelligence, physiological monitoring, and biomarker-based assessment may provide complementary information. However, their current clinical application remains limited by challenges in validation, feasibility, and implementation. Overall, the available evidence supports a multimodal approach that combines standardized assessment, evidence-based non-pharmacological analgesia, staff training, and cautious integration of digital decision-support tools in neonatal pain management.

The selection of pain assessment tools has to balance psychometric rigor with clinical practicality. Multidimensional assessment tools, such as the PIPP and NIPS, fit well with research and pain assessment in complex clinical settings when you need high precision. For routine bedside procedural pain assessment where speed and simplicity come first, the NIPS is a handy alternative, whereas the PIPP/PIPP—R may be preferred when gestational age adjustment is critical. Regular, relevant training is key to ensuring inter-rater reliability (50). In the future, mixing objective data coming from technologies such as facial expression analysis and physiological indicator monitoring with structured behavioral assessment scales could allow for more comprehensive and objective neonatal pain assessment.

Non-pharmacological analgesic strategies should be at the core of neonatal pain management protocols. Getting these strategies to work well depends on comprehensive institutional support, including the drawing up of specialized clinical practice guidelines,delivering professional training for healthcare staff, allocating resources, and the cultivation of a clinical culture that prioritizes pain prevention as a fundamental principle (49, 51). Getting parents involved in non-pharmacological analgesic interventions such as kangaroo care doesn't just help clinically—it also boosts parents’ sense of participation and initiative in caregiving.

### 4.1 Promise and hurdles of digital health solutions

Artificial intelligence and continuous monitoring technologies hold great potential in addressing the shortcomings of intermittent and subjective pain scoring. Future research and development should focus on building real-time monitoring systems with clinical transparency (i.e., explainability), which must be trained on diverse, high-quality datasets that accurately reflect the clinical reality of neonatal intensive care units (NICUs) (4, 39). The core objective of such research and development is to create intelligent decision-support tools that assist clinical judgment rather than replace clinical decision-making.

### 4.2 Clinical challenges

#### 4.2.1 Gaps between knowledge and clinical practice

Despite robust evidence-based support, the clinical implementation of routine pain assessment and non-pharmacological analgesic interventions remains inconsistent. Institutional barriers such as excessive workload, inadequate professional training, and the absence of standardized clinical protocols are the key factors contributing to this issue (50, 51).

#### 4.2.2 Limitations in the application scope of evidence-based evidence

The impact of specific non-pharmacological analgesic interventions on the long-term neurodevelopment of neonates has not yet been fully and clearly understood (6). In addition, evidence-based evidence regarding interventions for persistent neonatal pain and pain management in high-risk neonatal populations (e.g., infants with neonatal opioid withdrawal syndrome, NOWS) is still in the stage of gradual accumulation (52, 53).

#### 4.2.3 Dilemmas in the clinical translation of technological achievements

How to transform high-precision machine learning models into easy-to-operate, clinically accepted bedside devices has become a core challenge facing the current interdisciplinary field (4).

### 4.3 Limitation

This review has several limitations. First, the review was not prospectively registered, which may limit transparency and reproducibility. Second, only English-language publications were included; therefore, relevant studies published in other languages may have been missed. Third, because this was designed as an integrative literature review rather than a systematic review with meta-analysis, the included evidence was synthesized narratively. The studies varied substantially in population characteristics, gestational age, clinical settings, types of painful procedures, intervention components, comparators, outcome measures, and pain assessment scales. Therefore, formal statistical assessment of heterogeneity and quantitative meta-analysis were not performed. Fourth, although methodological limitations of the included studies were considered during synthesis, a formal risk-of-bias assessment using study-design-specific tools or a GRADE evaluation of the certainty of the evidence was not conducted. Finally, evidence for repeated or prolonged neonatal pain, high-risk neonatal populations, and real-world implementation of digital assessment tools remains limited, which restricts the strength of clinical recommendations in these areas.

## 5 Conclusion

This integrative review indicates that neonatal pain management should be based on standardized pain assessment and evidence-supported non-pharmacological analgesia. Validated multidimensional tools, such as PIPP and NIPS, remain useful for assessing neonatal pain, but their clinical value depends on consistent staff training, appropriate tool selection, and standardized implementation. Non-pharmacological interventions, including kangaroo care, breastfeeding or breast milk, oral sucrose or glucose for single procedures, non-nutritive sucking, facilitated tucking, swaddling, and combined multisensory strategies, can reduce procedural pain responses and should be incorporated into routine neonatal care whenever feasible. Emerging digital and objective assessment methods, particularly facial-expression-based artificial intelligence and physiological monitoring, may improve the objectivity and continuity of pain assessment. Still, they require further validation in real-world NICU settings before routine clinical use. Future studies should focus on improving implementation, evaluating repeated and combined interventions, clarifying long-term neurodevelopmental outcomes, and validating AI-assisted tools as clinical decision-support systems rather than replacements for professional judgment.

## Data Availability

The original contributions presented in the study are included in the article/Supplementary Material, further inquiries can be directed to the corresponding author.
